# Preparing Pharmacists for Collaborative/Integrated Health Settings

**DOI:** 10.3390/pharmacy7020047

**Published:** 2019-05-20

**Authors:** Frank J. Ascione

**Affiliations:** UM Center for Interprofessional Education, University of Michigan College of Pharmacy, Ann Arbor, MI 48109, USA; fascione@umich.edu; Tel.: +1-734-763-0100

**Keywords:** pharmacists, pharmacy education, interprofessional practice and education

## Abstract

Pharmacy practice is changing to accommodate the need for pharmacists to be better team members in newly emerging collaborative care and integrated health systems. Pharmacy schools could lead this change by educating students to be effective participants in these relatively new models of care. Schools are encouraged to follow the approach outlined in the recent guidance published by the Health Professions Accreditors Collaborative (HPAC) for interprofessional practice and education (“the new IPE”). This approach includes articulating an IPE plan, establishing goals, assessing student achievement of the necessary IPE competencies, developing educational plans that are multi-faceted and longitudinal, and modifying the existing assessment/evaluation process to ensure the quality of the IPE effort. These curricular decisions should be based on existing and new research on the effectiveness of IPE on student’s attitudes, knowledge, skills, and behavior. A key decision is how to create effective interactions between pharmacy students and those of other professions. Educational emphasis should be directed toward team building skills, not just individual competencies. The pharmacy faculty probably need to enhance their teaching abilities to accommodate this change, such as learning new technology (e.g., simulations, managing online exchanges) and demonstrating a willingness to teach students from other professions.

## 1. Introduction

An integrative/collaborative approach to health care services is needed to create a more efficient workforce and positive health outcomes [1,2]. Collaborative care is defined as occurring “when multiple health workers from different professional backgrounds provide comprehensive services by working with patients, their families, and communities to deliver the highest quality of care across settings.” Integrative Health Care is described as “bringing together inputs, delivery, management and organization of services related to diagnosis, treatment, care, rehabilitation and health promotion. Integration is a means to improve services in relation to access, quality, user satisfaction and efficiency.” Both approaches require enhanced cooperation among all levels of caregivers to ensure optimal service [1,3].

The call for more cooperation is not new and neither is the potential role of pharmacists in this effort [4,5]. Despite those historical appeals, there was limited local, national, and international effort to vigorously promote this approach for many years. That apathy began to change with the publication of the Institute of Medicine (IOM) report in 1999 entitled To Err Is Human [6]. This report suggested that as many as 98,000 hospital deaths per year were due to medical errors. A key finding of the report was the observation that these deaths likely arose “from the decentralized and fragmented nature of the health care delivery system—or “non-system,” to some observers.” 

The 1999 IOM report created a more concentrated effort of health professional collaboration, particularly around patient safety. However, other problems of the health care system, such as fragmented care, poor access, suboptimal patient outcomes, dissatisfied patients, and a frustrated work force, were still present. These issues were later highlighted in two key reports. The World Health Organization (WHO) noted in a 2010 report entitled that interprofessional collaboration connected with shared education would strengthen health care delivery and improve outcomes [1]. At the same time, an international commission of professional and academic leaders published a report in the Lancet calling for a new direction in health professional workforce education that emphasizes more interprofessional education and collaboration [7].

The result of these calls for action is an international movement toward reform of health professional education and practice [8,9,10]. This effort presents a great opportunity for pharmacy, which is recognized by many health professionals for its expertise in drug products and medication management [11,12,13]. However, pharmacy suffers the same barriers to team practice as other professions: a fractionated health system, scope of practice restrictions, hierarchical order of decision-making, and an insufficient educational focus on the skills needed to be an effective multi-professional team member or leader [14,15]. The purpose of this paper is to address the lack of educational focus and offer suggestions for curricular changes that support greater implementation of interprofessional practice and education (known as the “new IPE” [10]) principles in pharmacy education.

## 2. Approach

Pharmacy education traditionally focused on the “shaping” of student learners to become pharmacists. That identity revolves around teaching the skills needed to become drug product and medication management experts [16]. Like all health professions education, this “uni-professional” approach can be a barrier to preparing learners to function effectively in collaborative/integrative care practice settings, which could prevent them from providing high quality care to patients. Instead, a multi-professonial approach is needed with a shift toward inclusion of more interprofessional experiences, resulting in the development of a dual identity as both a pharmacist and as a key participant of collaborative care teams or as members of integrated health systems [9,17]. 

The idea of the dual or multi-professional approach of educating students to become pharmacists and effective members of the health care team has been discussed extensively in the past few years. The 2004 report on Educational Outcomes by the Center for the Advancement of Pharmacy Education (CAPE) mentions the need to educate pharmacy students to become effective members of interprofessional health care teams [18]. This focus was expanded in the 2013 CAPE guidelines which identified the “collaborator” role of pharmacy [19]. The 2013 report suggested that learning objectives for pharmacy students should include establishing a climate of shared values, defining clear roles and responsibilities, communicating effectively with other professions, and promoting joint problem solving. The 2013 CAPE report generated a number of research papers on desirable approaches to promoting IPE in pharmacy education in such areas as Integrative Health Education [20], Advanced Pharmacy Practice Experiences [21], and Entrustable Professional Activities [22]. 

The CAPE guidelines identified the need for IPE in pharmacy curricula and subsequent research identified possible strategies. These efforts were the basis for the Accreditation Council for Pharmacy Education (ACPE) Standard 11, which identifies key elements desired in a pharmacy curriculum to prepare students to become contributing members of an interprofessional team [23]. 

None of the efforts to date have provided a universally accepted plan for appropriate integration of IPE in pharmacy curricula. Fortunately, a recent guidance published by the Health Professions Accreditors Collaborative (HPAC), which is a collaboration among at least 25 of the accrediting agencies (including pharmacy), offers a template for developing that approach [9]. It suggests that every educational plan include the following characteristics:

- Rationale: Articulate a vision, framework, and justification for the IPE plan.
- Outcome-based Goals: State in terms that will allow the assessment of students’ achievement of objectives and interprofessional competencies for collaborative practice.
- Deliberate Design: Intentionally design and sequence a series of classroom, extracurricular, and clinical learning activities integrated into the existing professional curriculum that are longitudinal in nature, spanning the entire length of the program and including content and instructional formats appropriate to the level of the learner and to the outcome-based goals.
- Assessment and Evaluation: Use methods to assess individual learners’ mastery of interprofessional competencies and to evaluate the IPE plan for quality improvement purposes; and if appropriate, education and practice outcomes, research and scholarship.

These are general guidelines for all health professions educational programs. The specifics for each health profession have not been developed yet. However, possible applications to pharmacy education are offered in the following paragraphs: 

Rationale. Pharmacy educators have a solid rationale for the development of pharmacists as drug product and medication therapy experts, especially applied to areas such as improved therapeutic outcomes, patient adherence, drug safety, and transitions of care [24,25,26]. The expansion of this professional role to becoming effective participants in collaborative/integrative care is not as clear. The HPAC guidelines suggest that a conceptual model is needed to provide an overview of how collaborative activities are linked to better health outcomes. The example in the guidance is the Institute of Medicine Interprofessional Learning Continuum Model that illustrates the continuum of IPE through all levels of learners (undergraduate, graduate, and current practitioners) and how the learning process is affected by factors such as culture and the availability of needed resources [2]. Another useful model is the one developed by WHO, which suggests a positive relationship between IPE, collaborative care, and positive health outcomes [1]. 

These models imply that the pharmacist’s expert knowledge of drug products and medication management would be a necessary but not a sufficient factor in ensuring better health outcomes. To be more effective contributors, pharmacy education would need to go beyond teaching drug product/medication expertise to a more expansive instruction on factors such as the social determinants of health, the principles of human behavior, and non-medication strategies to improve health [19,27,28,29,30]. 

Outcome-based goals. Pharmacy educational programs already have a clear set of learning outcomes identified through the standards set ACPE and promoted by CAPE [19,23]. These outcomes include a strong foundational knowledge in the sciences, understanding of the principles of pharmacy practice, demonstration of the skills needed to manage medication therapy and medication use systems, and awareness of the best pathways to promoting health wellness in improving population-based care. 

The HPAC guidance suggests that a related but broader set of competencies are needed to effectively cooperate with other health professionals. The competencies created by the Interprofessional Education Collaborative (IPEC) [31] was the suggested starting point for dual interprofessional development of students. They are:

Competency 1, Values/Ethics for Interprofessional Practice: Work with individuals of other professions to maintain a climate of mutual respect and shared values. 

Competency 2, Roles/Responsibilities: Use the knowledge of one’s own role and those of other professions to appropriately assess and address the health care needs of patients and to promote and advance the health of populations. 

Competency 3, Interprofessional Communication: Communicate with patients, families, communities, and professionals in health and other fields in a responsive and responsible manner that supports a team approach to the promotion and maintenance of health and the prevention and treatment of disease. 

Competency 4, Teams and Teamwork: Apply relationship-building values and principles of team dynamics to perform effectively in different team roles to plan, deliver, and evaluate patient/population-centered care and population health programs and policies that are safe, timely, efficient, effective, and equitable.

ACPE has already addressed some of these competencies under its interprofessional education standard (standard 11). This standard focuses on demonstrating competence in three key elements: Interprofessional team dynamics, interprofessional team education, and interprofessional team practice [23]. The newness of the standard means that there are very few examples of successful strategies performed by Colleges of Pharmacy to meet those criteria. 

Deliberate Design. Pharmacy education programs already need to be intentionally designed and sequenced to meet accreditation [23] and licensing standards [32]. The design for IPE preparation would emphasize the same approach but would focus on a progression based, multifaceted, longitudinal integration of the IPEC interprofessional competencies into the full length of the existing pharmacy curriculum [31].

A key aspect of an IPE educational program is the focus on pharmacy students learning about, from, and with students from other professions regarding how to collaborate and improve health outcomes, which is consistent with the WHO definition of IPE [1]. In order to achieve this goal, traditional methods of teaching pharmacy students (e.g., lecture or classroom discussions) need to change. 

Approaches need to be multifaceted and include student-to-student IPE learning activities that go beyond courses, such as one-time gatherings of many different professions to address an issue (e.g., disaster preparedness events), online exchanges, and IPE simulations. Formally designed team based learning exercises with students from other professions need to be emphasized. Clinical rotations that highlight multi-professional teamwork supervised by faculty from multiple professions are needed to demonstrate the full value of collaborative care [28,33,34,35]. 

Assessment and Evaluation. Pharmacy accreditation already focuses on the key elements of student assessment, the quality of the mentoring process, and the value of the strategic educational plan [23]. IPE assessment builds on that foundation by focusing on student abilities to function as effective team members; emphasizing supervision and mentoring by a multi-professional faculty in team care environments; and strategic planning that includes the dual process of professional and interprofessional socialization. Assessment of the pharmacy student should include measures of changes in perceptions and knowledge of the capabilities of other professions, understanding of teamwork, and the demonstration/performance of collaborative behaviors in simulations and health practice settings [2,9,31].

## 3. Discussion

Pharmacy faculty are always confronted with challenges to update the curriculum to prepare students for the constantly changing health care environment. Moving toward a more interprofessional focus is especially challenging because of the need to engage other professional schools in the process. Here are some useful suggestions, based on my long time experience as a pharmacy educator (and Dean) and as a Director of a University-wide Interprofessional Center, that may assist in ensuring that the IPE curricular goals are met.

- The curricular decisions on IPE should be based on research that identifies the best educational strategies. This information is lacking in many areas because the IPE movement is still new, which means that more scholarship is required in this area. Many questions need to be resolved through thoughtful research and vigorous assessment of the various efforts. For example, what will be the typical practice models in the future and what would be the key roles of the various health professions? What are the specific skills needed to become an effective team member? Who are the important individuals that pharmacists need to interact with in the collaborative/integrative care environment? Does the nature of the pharmacist team role change in different practice settings (e.g., institutions vs ambulatory care)? What are the proper links between learner education, effective collaborative/integrative practice, and positive health outcomes? Resolution of these questions are needed before a valued based IPE curriculum can be successfully implemented.
- Despite our knowledge gaps, a crucial ingredient to any IPE curriculum is the level of discourse with students from other professions. The WHO definition of IPE emphasizes intense interaction of learners from different professions directed toward joint problem solving exercises [1]. The key for pharmacy curricular planners is deciding the priority of formal contact with a multitude of professions given the inherent logistical challenges. ACPE’s standard 11 states that the “curriculum prepares all students to provide entry-level, patient-centered care in a variety of practice settings as a contributing member of an interprofessional team. In the aggregate, team exposure includes prescribers as well as other healthcare professionals” [23]. This standard is helpful because it recognizes the medication management focus of pharmacy practice. However, it does not address the value of interactions with professionals who are not prescribers e.g., social work). These interactions should be based on their value to enhancing the perspective/knowledge of pharmacy students, not simply their educational accessibility. Co-location of these schools on the same campus is desirable but not necessary; it is appropriate to meet with students from other professions through specific collaborating institutions or through regional networks [9,36,37].
- The IPE emphasis should be on team building skills not just individual competencies [38]. The models used to support the rationale for interprofessional education emphasize the value of health professional teamwork in creating positive health outcomes [1,2,27]. Thus, popular team building techniques such as TeamSTepps [39] or the Interprofessional Collaborator Assessment Rubric (ICAR) [40] should be used extensively in the teaching process in order to emphasize group development. The focus is on educating pharmacy students to engage in shared decision making, joint accountability for patient care and population health [41].
- Pharmacy educators need to accept, endorse, and participate in a multi-professional approach to teaching their students. These actions may consist of acquiring a new set of teaching skills. These skills include effective facilitation of formal team based learning, use of technology such as simulations, or creating/managing online multi-professional forums. There also must be a willingness to teach non-pharmacy learners and to encourage other professionals to instruct pharmacy students [28,31,35,41,42].

## 4. Conclusions

Pharmacists are moving toward more active participation in collaborative/integrative models of care. This involvement emphasizes teamwork skills, more extensive interaction with other health professionals, and a broader understanding of the patient’s health needs. Implementing IPE in pharmacy education is necessary in order for students to be successful in this new practice model. The approach to implementation of IPE discussed in this paper is endorsed and supported in the HPAC Guidance [9]. However, implementation will only be effective if it is not viewed as a supplemental addition but rather a full integration into the curriculum. There are many examples of productive new attempts at curricular implementation in pharmacy schools [43,44,45,46,47]. These strategies are likely to expand in the future. 

The focus of this paper is on the implementation of IPE in the United States, but these academic and practice issues are prevalent throughout the world [48]. In fact, the WHO report promoting IPE viewed the movement as an important strategy to mitigate the global workforce crises [1]. The International Pharmaceutical Federation (FIP), the major international pharmacy organization, has promoted interprofessional education and practice for many years. Its 2016 report entitled: “Global Vision for Education and Workforce” emphasizes that pharmacy must develop a care model that is based on collaboration with other health professionals [49].

The need for pharmacists to be educated to be more collaborative is universal but the strategies are more likely to vary by country due to different pharmacy educational requirements, regulation of the scope of pharmacy practice, payer systems, and the availability of other health professionals [50,51]. Nevertheless, examples of successful implementation of IPE within pharmacy education and practice exist outside the United States, many of which set the standard for the whole world [52,53,54,55,56,57]. 

Successful integration globally of IPE principles into pharmacy curricula is extensive and will continue to grow in the future. These efforts will increase the educational shift necessary to ensure pharmacists are essential members of the future collaborative teams and full participants in the integrative health approaches needed to ensure better health outcomes to the patients we serve.
